# Elaboration of Silicon Nanostructures with Vapor-Phase Silver Assisted Chemical Etching: Correlation between Structural and Optical Properties

**DOI:** 10.3390/nano13101602

**Published:** 2023-05-10

**Authors:** Chohdi Amri, Shengzhong (Frank) Liu, Adel Najar

**Affiliations:** 1 Department of Physics, College of Science, United Arab Emirates University, Al Ain 15551, United Arab Emirates; chohdiamri@uaeu.ac.ae; 2 Laboratoire de Photovoltaïque, Centre de Recherches et des Technologies de l’Energie, Technopole de Borj Cedria, BP: 95, Hammam-Lif 2050, Tunisia; 3 Dalian National Laboratory for Clean Energy, iChEM, Dalian Institute of Chemical Physics, Chinese Academy of Sciences, Dalian 116023, China; 4 Key Laboratory of Applied Surface and Colloid Chemistry, Ministry of Education, Shaanxi Engineering Lab for Advanced Energy Technology, School of Materials Science and Engineering, Shaanxi Normal University, Xi’an 710119, China

**Keywords:** vapor phase-silver assisted chemical etching, porous silicon, photoluminescence, quantum confinement effect, oxide layer

## Abstract

Based on the widely used wet metal-assisted electroless etching, we develop in this work a novel vapor-phase silver-assisted chemical etching (VP-Ag-ACE) suitable for the elaboration of highly doped p-silicon (Si) nanostructures with strong, visible, and multi-peak photoluminescence (PL) emissions. The lateral and vertical etching rates (LER and VER) were discussed based on the etching mechanism of the VP-Ag-ACE. The antireflective suitability of the vapor-etched layer has been evaluated by a reflectivity measurement and exhibits reflectivity values lower than 3%. The PL emission at both room and low temperatures emissions were deeply discussed and correlated with the structural properties of the Si morphologies and their surface states based on the FTIR results.

## 1. Introduction

Bulk silicon (Si) exhibits restrict applications in the optoelectronic filed mainly resulting from its indirect band-gap structure. In 1990, Canham discovered the photoluminescence (PL) emission at room temperature from porous silicon (PS), following that, enormous worldwide efforts have been focused on exploring both elaboration methods and PL origins of the PS making it a block building in optoelectronic and photonic industries [1,2,3]. The elaboration of the PS in these sophisticated technologies is mainly based on chemical and top-down techniques including electrochemical anodization (ECA), stain etching (SE), chemical vapor etching (CVE), and the liquid phase metal-assisted chemical etching (MACE). The ECA method employs a platinum wire as cathode and Si substrates as anode immersed in a mixture of HF acid and electrolyte under the application of an external bias. The external bias is applied to result in a homogenous porous layer. In the SE method, PS layers are produced by immersing the Si substrates in the H_2_O/HF/HNO_3_ mixture [4]. While for the CVE, a highly luminescent porous layer is formed by exposing Si substrates to HF/HNO_3_ vapors [5]. On the other hand, the MACE method requires the deposition of the metallic catalyst on the Si surface followed by chemical etching in a mixture of HF and an oxidant [6]. Recently, the last two methods were deeply studied in both experimental and theoretical fields due to their simplicity, rapidity, and productibility in the elaboration of highly luminescent Si nanostructures [7]. By combining the last two techniques, Owen et al. developed the vapor phase-MACE (VP-MACE) method where the catalytic metals (Au, Ag, and Pd/Au) were deposited on the Si surface and then exposed to the vapors emanating from HF/H_2_O_2_ hot solution [8]. Different 3D Si nanostructures have been produced. Later, Amri et al. successfully elaborated a combination of microcavities and sporadic Si nanowires (Si NWs) by using the vapor phase-silver-assisted chemical etching (VP-Ag-ACE) for their application in photovoltaic cells. A reasonable model of the etching mechanism has been proposed [9]. Recently, Kubota et al. reported the elaboration of PS using the VP method but using graphene oxide as a catalyst on the top of Si substrates [10]. To the best of our knowledge, these previous works discussed only the structural properties of Si nanostructure and the etching mechanism of the VP-MACE and abandoned the investigation of their optical properties especially the discussion of the PL origin. The luminescent property of the PS layer makes it as an excellent candidate for potential applications in optoelectronics and photonics [11,12]. Nevertheless, the PL mechanism from PS is still under intense debate due to the many factors that govern the visible emission, in particular, the quantum confinement effect (QCE) within the size-reduced Si nanocrystals (Si NCs), the oxide- and/or the hydrogen-terminated nanostructures, the localized state, surface/defect states in the oxide, etc. [13,14]. Furthermore, aiming to discuss and understand the PL mechanism from PS structures, temperature-dependent PL has been performed by several groups. The results of these studies are miscellaneous and sometimes seem to be contradictory especially when the PS spectrum presents multi-peaks in the visible range where various PL origins are interfered in the luminescent mechanism.

In this investigative work, we focus our attention on the elaboration of the luminescent PS layer via the VP-Ag-ACE and the discussion of the PL origin. Different PS morphologies are obtained by varying the etching duration. The total reflectivity of the surface exhibit low values (less than 3%) at the visible spectral range. The room temperature PL spectra exhibits three distinct peaks and are attributed to different origins and luminescent mechanisms. Based on the temperature-dependent PL and the FTIR results, a clear discussion of the PL mechanism has been proposed.

## 2. Materials and Methods

The Si substrates used here are p-type with (100) orientation, with 1 cm^2^ surface area, 280 μm thickness, and a resistivity ranging from 0.015 to 0.0018 Ω·cm. Prior to all, Si substrates are immersed in CP_4_ acid solution, a mixture of (HNO_3_: 64%, HF: 16%, CH_3_COOH: 20%) for 30 s to remove any trace of contamination. The elaboration process of PS using the VP-Ag-ACE method follows two steps. First, Ag nanoparticles (Ag NPs) are plated onto the Si surface by dipping in a mixture of 4.8 M HF and 0.001 M AgNO_3_ for 1 min. The second step consists in exposing the Ag NPs led Si substrates to the vapors of HF/H_2_O_2_ solution with a volume ratio of 4/1 and heated at 70 °C for an etching duration varying from 5 min to 100 min. The etching solution is renewed after each sample to avoid the aging of the solution. Thereafter, etching samples were rinsed with deionized water and immersed in HNO_3_ solution for several minutes to remove the Ag NPs, rinsed and finally dried with N_2_ flux.

The structural property of the PS layers was investigated with the scanning electron microscope (SEM, HITACHI 4800). PL spectra were recorded at both room and low temperatures under the excitation of 267 nm laser wavelengths. The reflectivity of samples was measured by LAMBDA 950 de Perkin Elmer UV/Vis/NIR Spectrophotometer in the 250–1100 nm wavelength range. The FTIR spectra were recorded using Nicolet MAGNA-IR 560 Spectrometer with a resolution of 2 cm^−1^.

## 3. Results and Discussion

### 3.1. SEM Analysis

**Figure 1:**
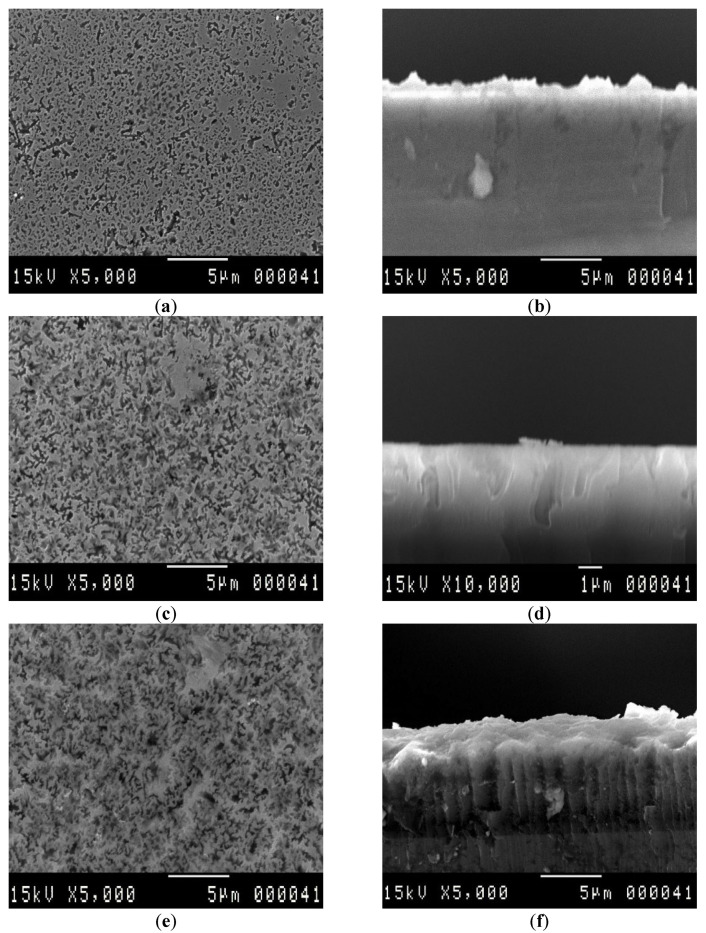

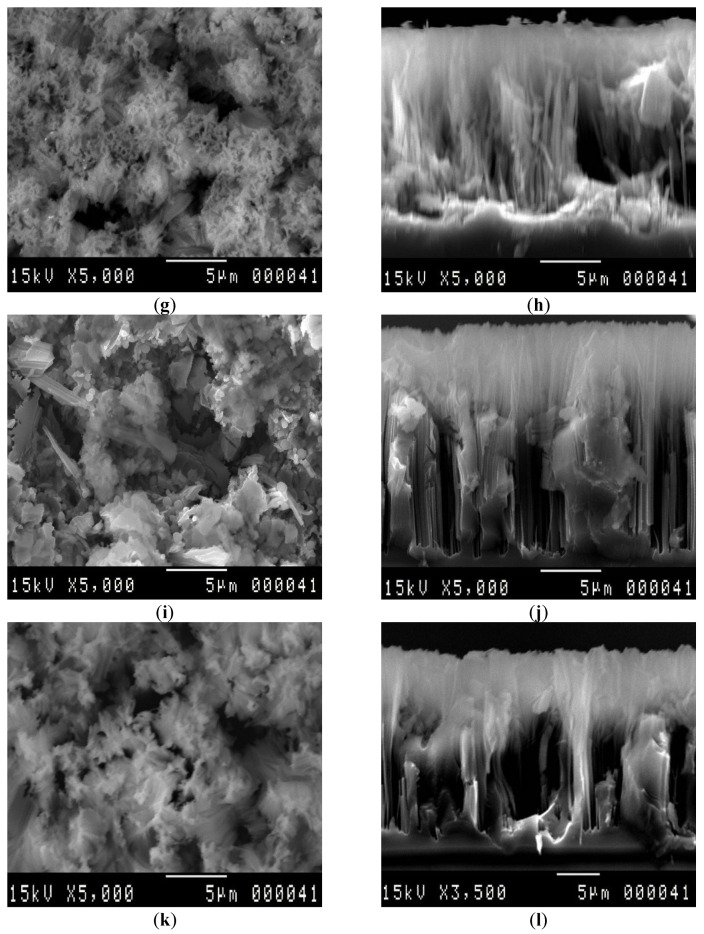
Top-view and cross-section SEM images of PS elaborated via VP-Ag-ACE during (**a**,**b**) 5 min, (**c**,**d**) 20 min, (**e**,**f**) 40 min, (**g**,**h**) 60 min (**i**,**j**) 80 min, and (**k**,**l**) 100 min.

Top-view and cross-section SEM micrographs of PS samples treated with VP-Ag-ACE for different exposure durations ranging from 5 min to 100 min are displayed in Figure 1. After 5 min of etching, we observe in Figure 1a,b the appearance of star-like tiny holes distributed uniformly on the whole surface. The cross-section image indicates that the Si etching follows preferentially the vertical direction with a non-uniform etching rate where the maximum depth is nearly about 0.5 µm. As the etching duration increases, the pore diameter is enlarged, resulting from the opening between the neighbor pores to exhibit a sponge-like structure. The thickness of the porous layer remains in increase, and we observe a new inclined etching path leading to a non-uniform vertical etching rate (VER). Exceeding 60 min of etching, the pore diameters continue their expansion, and the Si skeleton exhibits a star-like structure instead of the sponge-like structure. Thereafter, this structure is dissolved and replaced by a grain-like structure which is clearly observed at 80 min. On the other hand, the vertical etching depth is still increasing to reach its maximum of 18 µm at 100 min. We note that the vertical etching becomes more uniform, and the porous layer exhibits a Si NWs-like structure. However, for long etching duration, the ordered Si nanostructure seems to be dissolved at their bottom where large holes are formed as shown in Figure 1l.

Table 1 summarizes the structural characteristics of the PS elaborated with VP-Ag-ACE. As we mentioned previously, the lateral and vertical etching rates (LER) are increasing with the etching duration. The pore diameter and the thickness increase from 0.5 µm and 0.5 µm at 5 min to reach 5 µm and 18 µm at 100 min, respectively. However, the LER and VER are not uniform. For low etching durations between 5 and 60 min, the LER decreases from 0.1 µm/min to 0.05 µm/min while the VER is linearly increasing from 0.1 µm/min to 0.2 µm/min. As a result, a more ordered vertical structure is formed and seems to be a nanowire-like structure. For a high duration between 60 and 100 min, the LER is fixed around 0.05 µm/min while the VER starts to decrease from 0.2 to 0.18 µm/min accompanied by destruction of the ordered structures and the formation of microcavities at their bottom. These observations can be explained based on the VP-Ag-ACE mechanism. As the etching duration increases while keeping all the other parameters fixed, microscopic HF/H_2_O_2_ droplets condense on the top of the Ag NPs due to the temperature gradient between the Si surface and the hot vapors [15]. At this moment, the reduction of the H_2_O_2_ molecules at the Ag NPs’ top surface leads to the transfer of two holes (h^+^) to the Si volume through the Ag NPs forming a thin SiO_2_ layer beneath the Ag NPs which will be dissolved under the effect of the HF condensed acid droplets, resulting in the elaboration of the observed structures. As the etching duration increases, the Ag NPs groove deeply into to Si substrate under the effect of the electric field ($\vec{E}$) pointing from the positively charged Si to the negatively charged Ag NPs [6]. In addition, the increase of the exposure duration to the hot acid steam increases the substrate temperature and the condensed droplet amount. As a result, the oxidation/dissolution rate of Si atoms is enhanced, leading to the increase in the LER and VER. However, for long etching durations between 60 and 100 min, the excess of injected holes, resulting from the increase in the substrate temperature and the amount of condensed micro-droplets migrate to the Ag NPs sidewalls, resulting in the decrease in the VER and the destruction of the porous structure at their bottoms where the Ag NPs are located after etching.

**Figure 2:**
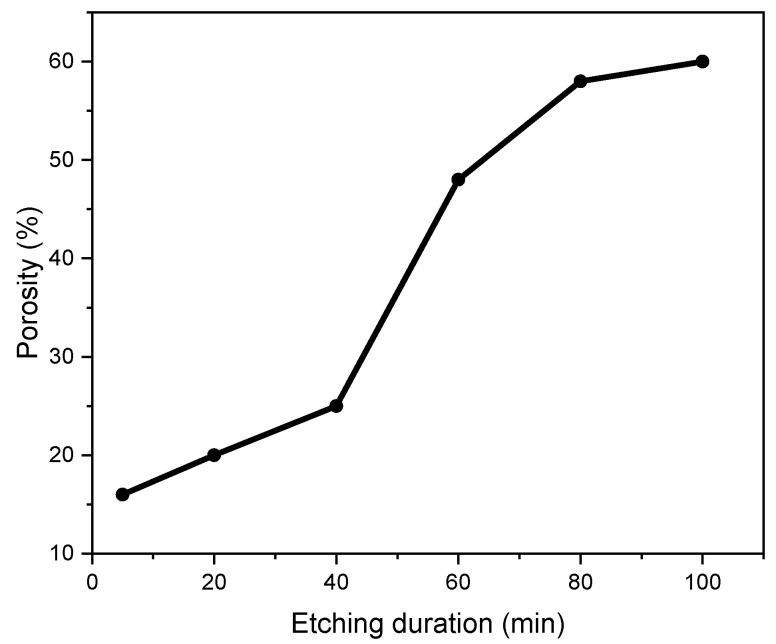
Evolution of the PS porosity with the etching duration.

To further investigate the PS morphological properties, the porosity of the samples with the etching duration is calculated using a gravimetric method based on Equation (1) and shown in Figure 2 [16]:

(1) $$P\;\left(\%\right)=\frac{m_{1}-m_{2}}{m_{1}+m_{3}}\times 100$$

$m_{1}\;$ and $m_{2}\;$ are the masses of Si before and after the formation of the PS layer. $m_{3}\;$ is the mass of Si substrate after the removal of PS layer by immersing in a 1 M NaOH. The porosity of the PS samples increases from 16.6% to reach 61% as the duration increases from 5 min to 100 min. In the literature, the increase in the porosity is attributed to the increase in the etching duration governed by the doping type and level (n or p-type and the resistivity), the composition of the etching solution, the etching time, the temperature, the illumination, etc. [17]. In our case, the increase in the porosity from 16.6% to reach 61% is attributed to the high-etching temperature and the low resistivity of the p-type Si substrates. The variation of the porosity with the etching duration will be discussed further and correlated with the PL properties of the PS samples.

### 3.2. Total Reflectivity

**Figure 3:**
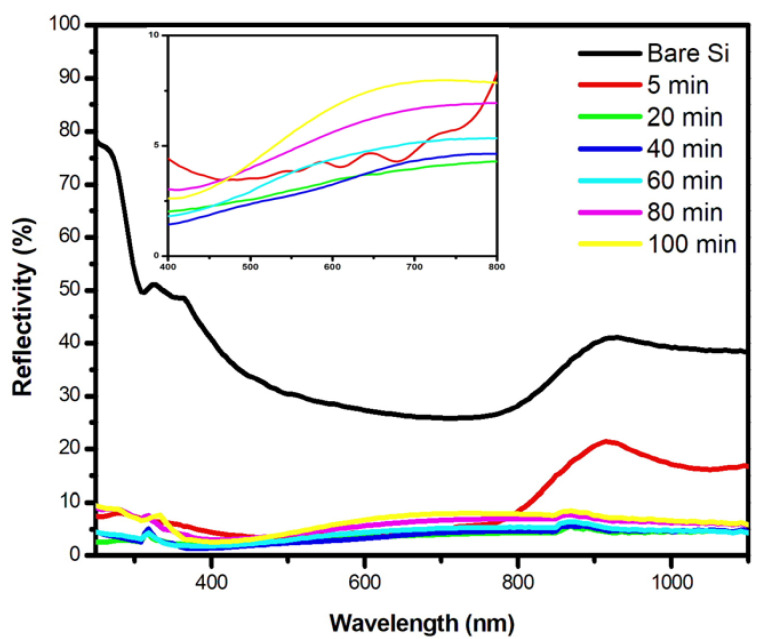
Total reflectivity of PS samples elaborated via VP-Ag-ACE at different etching durations. The insert shows the reflectivity in 400–800 nm wavelength range.

To investigate the effectiveness of the PS layer elaborated with VP-Ag-ACE method in the photovoltaic application, we carried out in Figure 3 the total reflectivity of five different samples in the 250–1100 nm spectral range. A non-treated sample (bare Si) was taken as a reference. As it is well known, Si substrate with a smooth surface exhibits a relatively high surface reflectivity, about 30% in the visible range. After VP-Ag-ACE, a considerable decrease in the reflectivity to less than 8% is observed for all the samples even for a short etching time (5 min). This behavior is explained by the formation of a thin-porous layer acting as a light-trapping layer through the multiple internal reflections of the incident light between the PS sidewalls. As the etching duration increases without exceeding 60 min, the total reflectivity still decreases to reach its minimum value of about 3% in the visible range as shown in the insert of Figure 3. This observation is explained by the formation of nanopores coating the thicker PS layer leading, in addition to the multiple reflection of the incident light, to the increase in the refractive index with depth and the enhancement of the surface rugosity [18]. However, when exceeding 60 min, the total reflectivity of the surface gradually increases reaching 8% after 100 min of etching. We attribute this observation to the expansion of the pore diameters to reach 5 µm and the degradation of the PS bottom layer forming an overlapped structure as shown in Figure 1k,l limiting, as a result, the light scattering on the pore sidewalls. It is noteworthy that the reflectivity values of our samples obtained using VP-Ag-ACE are less than the previously reported values in the following references [9,19], which prove the effectiveness of the PS layer elaborated with VP-Ag-ACE for photovoltaic applications.

### 3.3. PL Spectroscopic Analysis

**Figure 4:**
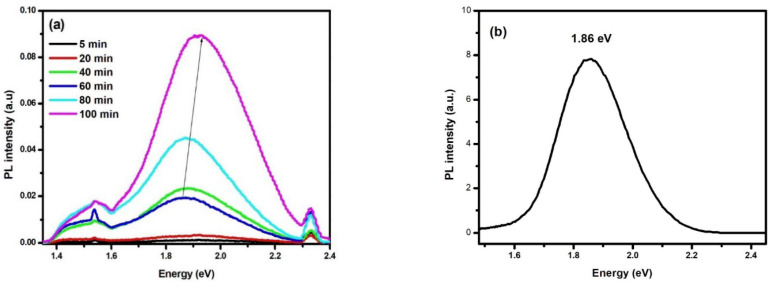
(**a**) PL emission from PS elaborated with VP-Ag-ACE at different etching durations. (**b**) PL emission from p-Si NWs prepared with wet Ag-ACE in HF/H_2_O_2_ solution.

PS layer is a distinguished material due to its interesting biodegradability and mechanical and thermal properties which makes it a leader in microelectronic applications [20]. Furthermore, following the discovery of the visible PL emission from PS at room temperature by Canham [1] great efforts from the scientific community have been carried out to discuss the PL origin, its properties, and applications [5,21]. Dealing with the same framework, we discuss the effect of the VP-Ag-ACE duration on the PL emission from PS layers, aiming to propose a comprehensive model to understand the origin of this interesting emission. Figure 4a shows the PL emission of the PS sample under 266 nm laser excitation wavelength. For the short etching durations between 5 and 20 min, no significant PL emission was recorded which confirms our previous results that these samples have low porosities (16.6% and 20%, respectively) and behave like the bulk Si with an indirect bandgap. As the etching duration exceeds 40 min, PS exhibits three distinct Gaussian PL peaks. These peaks are red-near infrared peaks centered on 1.54 eV, broad and high-intensity red-emission peaks centered on 1.87 eV, and green-emission centered on 2.33 eV (corresponding wavelength 805 nm, 663 nm, and 532 nm, respectively). These peaks are named first, second, and third peaks, respectively. As the etching duration increases from 40 min to 100 min, two main results are obtained. The first is the steady increase in the PL intensity of the three peaks where the red PL peak is more enhanced. The second observation is the shift of this red emission toward the higher energy by 0.052 eV reaching 1.92 eV. However, the peaks centered on 1.54 eV and 2.33 eV remain fixed at their position even the etching duration increases. We note that this broad-band PL emission shows nearly five times the intensity of the lowest energy peak and seven times the intensity of the highest energy peak. To confirm the appearance of the three PL emissions from PS is related to the etching method and the PS layer morphologies not to the intrinsic Si properties, we carried out in Figure 4b the PL emission of porous Si NWs (p-Si NWs) elaborated with the wet Ag-ACE method in HF/H_2_O_2_ solution and using the same substrate as a starting material. The PL peak was recorded at the same conditions as the PS samples. We observe a wide and intense Gaussian red emission centered on 1.86 eV with the total absence of the two PL shoulders around 1.54 eV and 2.33 eV. This observation excludes the hypothesis that PL originates from the defects in the Si substrate and confirms our assumption that PL emissions are related to the elaboration technique and the properties of the PS layers. The appearance of three PL peak emissions indicates that different mechanisms are involved in the radiative transitions in PS nanostructures. To unveil the origin of the PL emission of the individual contributions of the different possible transition processes, the next section discusses the temperature-dependent PL spectra of the PS etched for 100 min in the range of 20 to 300 K.

In the literature, the PL origin of the two [22,23,24] or three separated peak emissions from PS or p-Si NWs was reported with different explanations. Using electrochemical anodization, Ray et al. reported three distinct peaks located on 775–800 nm, 550 nm and 425~468, for current density J = 80 mA/cm^2^ [25]. Based on the temperature dependent PL characteristics of PS, they concluded that these peaks originate from two competitive processes involving band-to-band and oxide related interface mediated transitions.

Using the wet Ag-ACE, Iatsunskyi et al. reported that the PL spectrum of PS can be deconvoluted into three peaks located at 480, 550, and 670 nm (corresponding energies 2.58, 2.25, and 1.85 eV) [26]. They attributed the red PL peak (1.85 eV peak) to the QCE within the small Si NCs. The PL peak at the green region (2.25 eV peak) is associated with the formation of AgF or AgF_2_. However, the blue PL peak localized at 480 nm (2.58 eV) was not discussed. On the other hand, Parida et al. reported the emission of three separated PL peaks from p-Si NWs elaborated on micro-pyramids [27]. These peaks are located at 419 nm (2.95 eV), 507 nm (2.44 eV), and 637 nm (1.94 eV) for a 15 min etching. They attributed the appearance of these emissions to the high porosity of the nanostructures leading to the confinement of charge carriers in Si NCs. However, they neglected the interpretation of each peak.

**Figure 5:**
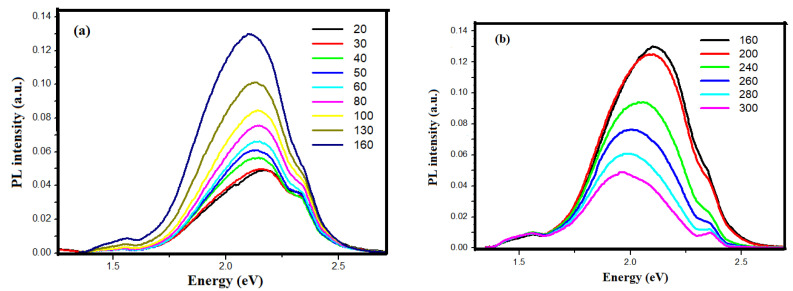
Temperature-dependent PL spectra of the PS etched for 100 min in the range of (**a**) 20 to 160 K and (**b**) in the range 160 to 300 K.

Figure 5 shows the temperature-dependent PL spectra of the PS etched for 100 min in the range of 20 to 160 K (Figure 5a) and the range of 160 to 300 K (Figure 5b). At first sight, the most intense PL peak dominates the spectra and monotonously shifts toward the lower energies from 2.17 eV to 1.9 eV as the temperature increases from 20 to 300 K. Similar behavior was reported in many references and attributed to the shrinkage of bandgap at low temperatures and the enhancement of the electron–phonon coupling at elevated temperature which is in accordance with the Varshni model inducing a blueshift with the temperature decrease [21,22,23,25]. Based on that, we attribute the second PL peak to the QCE within the small Si NCs. Whereas the two other PL peaks are independent of the temperature and remain fixed in their initial positions 1.54 eV and 2.33 eV, respectively, which urged the authors to propose a different origin for these peaks.

On the other hand, the intensity of the dominant PL peak (2nd peak) varies as a function of the temperature. In the range between 20 to 160 K, a considerable enhancement of the PL intensity is obtained. However, as the temperature exceeds 160 K, a steady decrease in intensity is obtained. We can explain these results by the activation of the non-radiative recombination centers at relatively high temperatures, (≥160 K) resulting in the thermal quenching of the PL intensity. During this process, the photogenerated carriers reach the nonradiative centers, such as dangling bonds at the surface, more easily leading to the decrease in PL intensity with temperature increase.

However, for the first and the third peaks localized at 1.54 eV and 2.33 eV, their relative intensities increase as a function of the temperature until 160 K. Besides 160 K, the intensity of the first peak is stabilized and shows nearly the same intensity and shape where the intensity of the third peak is reduced showing a separate peak at room temperature without a significant change in their peak positions as mentioned above. These observations are different from the QCE prediction and within the Si NCs and seem to be contradictive with the Varshni equation relating the PL blueshift with the decrease in the temperature. Based on that, a different origin should be adapted to explain the origin of these peaks. Regarding the first peak (low energy peak), different mechanisms have been proposed and sometimes controversial. Lee et al. reported that PS without any post-treatment exhibits a broad red PL centered on 1.65 eV and attributed to the localized exciton at the interface region between Si NCs and SiO_2_ thin layer [28]. Zhang et al. reported that the 800 nm PL peak originates from the localized state related to Si-O bonds, and the self-trapped excitations in the Si NCs are the source of the PL emission [24]. Podhorodecki et al. reported the strong emission band centered at around 1.6 eV (S-band) is related to the recombination via the surface states of the Si NCs [29]. However, these papers investigated the origin of the red/NIR PL emission where it is the unique and the most intense peak. In case this peak is the weakest and related to other higher energy peaks, its origin is attributed to radiative recombination. During this process, a hole is relaxed at the interface states above the valence band or an electron is captured (or relaxed) at the interface states near the conduction band edge. Compared to band-to-band recombination, this low-probability process leads to lower energy PL peak in the red/NIR emission (800 nm). In addition, the relaxation of electrons through the interface states is less probable than that of holes [25]. This model proposed by Ray is the most adapted to explain our 1.54 eV peak. It was reported in the literature that the green PL emission (third peak) from PS is mainly originating from the oxygen vacancies in SiO_x_ which induce a very strong and anisotropic lattice distortion layer on the surface of the Si NCs [29,30,31].

By using an excitation wavelength of 267 nm, our results agree with the assumption of Ray et al. affirming that the visible PL from SiO_2_ is only detected when the excitation energy required to obtain is usually much greater than 325 nm [25]. 

**Figure 6:**
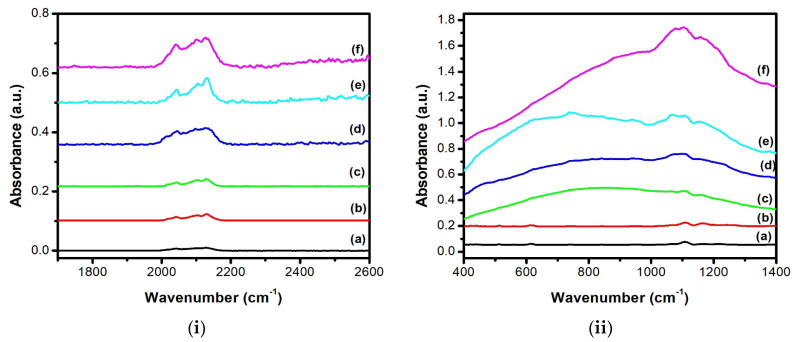
FTIR spectra of PS elaborated with VP-Ag-ACE during (a) 5 min, (b) 20 min, (c) 40 min, (d) 60 min, (e) 80 min, and (f) 100 min in (**i**) 400–1400 cm^−1^ and (**ii**) 1800–2500 cm^−1^ spectral range.

To further confirm our conclusion regarding the PL origin of three peaks, FTIR spectra of the five samples were carried out in (i) 400–1400 cm^−1^ and (ii) 1800–2500 cm^−1^ spectral range as shown in Figure 6. The main absorption bonds recorded in Figure 6i are localized between 1050–1180 cm^−1^ and attributed to the stretching modes of the Si-O-Si bonds. The presence of the oxygen-related peaks supports our suggestion about the PL origin of the first and third peaks which is related to the relaxation of the hole at the interface states related to Si-O bonds above the valence band, or the capture of an electron at the interface states near the conduction band edge and to the oxygen vacancies in SiO_x_ on the surface of the Si NCs, respectively. In addition, the absence of any absorption peak below 700 cm^−1^, usually attributed to the presence of metal bonds, excludes the hypothesis of Iatsunskyi et al. suggesting that the green PL peak is associated with the formation of AgF or AgF_2_ [26]. Figure 6ii shows the appearance of the SiH_x_ bonds in the stretching mode and localized between 2050 and 2200 cm^−1^. The appearance of these absorption peaks indicates the formation of the Si NCs within the PS layer and is responsible for the high-intensity red-emission peak centered on 1.87 eV through the QCE. The intensity of these peaks increases as a function of the etching duration indicating the increase in the luminescent center density which agrees with the PL observation. 

## 4. Conclusions

In this paper, PS layers were grooved on p-type c-Si wafers with a resistivity ranging from 0.015 to 0.0018 Ω·cm using the VP-Ag-ACE method. The effect of etching duration on the structural and optical properties of the PS has been studied. SEM micrographs show the formation of thick and expanded microcavities at long etching durations with an LER and VER of about 0.05 and 0.18 µm/min, respectively. The porosity of the samples varies from 16.6% to 61% and is attributed to the high etching temperature and the low resistivity of the substrates. The as-prepared PS layers exhibit low surface reflectivity (lower than 3%) suitable for photovoltaic applications. Based on the temperature-dependent PL spectra recorded under UV excitation, three distinct peaks localized on 154 eV, 187 eV, and 2.33 eV are obtained and attributed to the capture of the charge carriers at the interface states near the conduction band edge, the QCE within the size reduced Si NCs, and the oxygen vacancies in SiO_x_, respectively.

## Figures and Tables

**Table 1:** Evolution of the structural characteristics of the PS with the etching duration.

Etching Duration (min)	Pore Diameter (µm)	Pore Thickness (µm)	LER (µm/min)	VER (µm/min)
5	0.5	0.5	0.1	0.1
20	1	3	0.05	0.15
40	1.5	7	0.0375	0.175
60	3	12	0.05	0.2
80	4	15	0.05	0.187
100	5	18	0.05	0.18

## Data Availability

Not applicable.
